# Fracture Tables

**Published:** 1853-09

**Authors:** 


### EDITORIAL.

*Fracture Tables*, by FRANK H. HAMILTON, A. M., M. D., Prof. of Surgery in the University of Buffalo; accompanied with a supplement compiled from Dr. Hamilton's notes. By JOHN BOARDMAN, A. B.

THESE tables do not admit of any abridgement or analysis: we present the following from the supplement.

SUMMARY OF BONES OF SKULL.—I have taken the liberty of forming a summary from the valuable article "Lente's Statistics of Fracture of the *Cranium*," published January last in the "New York Journal of Medicine and the Collateral Sciences." In those tables *one hundred and twenty-eight* cases are reported, of which the result in *three* cases was not given, and in *eight* others the age was wanting; on this account I have omitted these in my summary of his tables. These results differ from those obtained from Dr. Hamilton's notes, but the reader, if he examines the cases in both tables will perceive that those recorded by Dr. Lente are such as have been brought into the *New York Hospital* during the last twelve years, the result of *recent* injuries, and that the proportion of those under *fifteen* years of age is very small compared to the other ages, while in Dr. Hamilton's notes more than one half occurred in the *first fifteen* years of life; also Dr. Hamilton's notes are *in part* composed of cases, which have come under his eye, after the lapse of several years from the time of the accident, and they cannot therefore so faithfully represent the average result of such injuries, since none but survivors are accounted for.

In a medico-legal point of view, the following suggestion of Dr. Lente, in the same article, is of value: in no case did death follow the receipt of the injury until after the lapse of some hours, even in the most desperate cases; nor does it appear possible for an ordinary blow upon the head, producing fracture of the skull, to cause death." In a recent criminal trial of great interest, it will be recollected that, at one stage of the proceedings, it was much discussed whether a blow upon the head with an ordinary weapon capable of inflicting death, could produce this result instantaneously; many eminent surgeons were examined, and the general impression was that the thing was exceedingly improbable, if not impossible, and the question was thus decided." A suggestion, that the reader will see is equally applicable to the notes of the thirty-three cases by Dr. Hamilton.

In the March number of the same Journal, Stephen Smith. M.

D., in an article entitled "Smith on Surgical treatment of Epilepsy," exhibits one of the frequent results of fractures of the cranium, as illustrated also by Dr. Hamilton's tables. In twenty-seven cases of epilepsy reported by Smith, twenty-four were known to have resulted from fracture of the bones of the head.

The New York Journal, before alluded to, vol. iii, 1840, speaks in very high terms of "Remarks on Fractures, by A. L. Pierson, M. D., of Salem, Mass.," which contains not only remarks, but also tables "drawn from the records of the Mass. General Hospital," "of all the cases of fracture ever treated in that institution." Also the Boston Med. and Surg. Jour., Aug., 1840, makes honorable mention of those tables. I regret that I have not had the opportunity of examining them.

In the same Journal, vol. iv., page 437, is a short article entitled "Cases of Fracture in the General Hospital of Hamburgh, during the year 1838, by Fricke." "The whole number treated was *seventy-two.* Of these, forty-two were cured, ten died, and twenty remained under treatment." Between 10 and 30 years there were twenty fractures; from 30 to 50 years, twenty-four fractures; 50 to 70 years, twenty-two fractures; 70 to 90 years, six fractures. Also the tibia and fibula were broken *twenty-two* times, the femor *seventeen* times, os brachii *seven*, and the clavicle *three* times—the *shortest term of treatment* was for the os nasi, *nineteen days*; and the *longest* for the neck of os femoris, *one hundred thirty-nine days.*

The author states that of the seventy-two, six remained from the last year; of the sixty-six who entered, fifteen were females.

Lente, N. Y. Jour., vol. iii, page 167, gives a statement of 1548 fractures, of which 1366 were males, and 182 females. I have not been able to determine the relative proportion in which fractures occur of the sexes: these, being the only statements I have been able to find on this point.

The only records I have found of the number of fractures drawn from a body of men, were the books of the Auburn State Prison. They show that of *five hundred and eighty* convicts admitted, *one hundred and twenty* reported at time of admission, ancient fractures.

The subject of *results*, which occupies the most prominent place in Dr. Hamilton's tables, is not considered in Fricke's table, nor am I informed that it constitutes a point in Pierson's tables, or in any others hitherto constructed. I am at least quite certain that by no one else, have the results been determined by measurements.

"Lente's Statistics of Fractures" published in the September number of the N. Y. Journal, give the "age, sex, season, and seat of fracture, &c.," in those fractures occurring in the N. Y. Hospital for the last twelve years, but in his tables, he makes no mention of the amount of shortening in the different fractures. To show the relative frequency of fractures of the different bones, he institutes "a comparison with the statistics of Malgaigne, at the Hotel Dieu of Paris, Lonsdale, at the Middlesex Hospital of London," and Norris, at Pennsylvania Hospital of Philadelphia; from which, as well as from Fricke, at the General Hospital at Hamburgh, and Dr. Hamilton's notes, I shall make the following abridgment by way of comparison.

| NAME OF BONES. | N. Y. Hospital. | Pennsylvania Hospital | Hotel Dieu. | Middlesex Hospital. | General Ho pital at Hamburgh. | Dr. Hamilton. |
|---|---|---|---|---|---|---|
| Thigh, . . . . . . . . . . . . . | 280 | 134 | 308 | 181 | 17 | 73 |
| Tibia and Fibula, . . . . . . | 442 | | 515 | 197 | 22 | 73 |
| Tibia, . . . . . . . . . . . . . . | 45 | 293 | 29 | 41 | 5 | 19 |
| Fibula, . . . . . . . . . . . . . | 92 | | 108 | 51 | 4 | 16 |
| Arm, . . . . . . . . . . . . . . | 161 | | 310 | 118 | 7 | 38 |
| Radius and Ulna, . . . . . . | 90 | 227 | 107 | 93 | 2 | 34 |
| Ulna. . . . . . . . . . . . . . . | 36 | | 29 | 64 | .. | 23 |
| Radius, . . . . . . . . . . . . . | 143 | | 160 | 197 | 1 | 27 |
| Clavicle, . . . . . . . . . . . . | 158 | 84 | 225 | 273 | 3 | 41 |
| Inf. Maxilla, . . . . . . . . . | 65 | 19 | 27 | 32 | 1 | 13 |
| Pelvis, . . . . . . . . . . . . . | 23 | 6 | 7 | 7 | 3 | 2 |
| Patella, . . . . . . . . . . . . | 30 | 16 | 45 | 38 | 1 | 7 |
| Scapula, . . . . . . . . . . . . | 17 | 10 | 4 | 18 | .. | 3 |
| Sternum, . . . . . . . . . . . . | 12 | 5 | 1 | 2 | 1 | 1 |
| Skull, . . . . . . . . . . . . . | 128 | 46 | 53 | 48 | .. | 33 |
| Amount, . . . . . . | 1722 | 840 | 1939 | 1392 | 67 | 403 |

It is thus by comparison of the cases occurring both here and elsewhere, that I have endeavored to arrive at the general truths, which can only be obtained from facts collected from various sources. It is by comparison of the *results* in the tables first published by Dr. Hamilton, with those I have compiled from his notes, and also by comparing both with other tables, that I have sought to prove their accuracy as statistical records. There will be seen a striking similarity in the average results between the tables in the case of almost every bone; the relative frequency of fracture being nearly identical, as stated by the different writers. It is true the statistics of Middlesex Hospital present a much larger proportion of fractures of the superior extremities than the other tables; but when it is ascertained that the number of beds in this Hospital is limited, and that a large proportion of those treated are "out-door patients," this disagreement will find a sufficient explanation.

